# Predicting couple therapy outcomes based on speech acoustic features

**DOI:** 10.1371/journal.pone.0185123

**Published:** 2017-09-21

**Authors:** Md Nasir, Brian Robert Baucom, Panayiotis Georgiou, Shrikanth Narayanan

**Affiliations:** 1 Department of Electrical Engineering, University of Southern California, Los Angeles, United States of America; 2 Department of Psychology, University of Utah, Salt Lake City, Utah, United States of America; University of Kent, UNITED KINGDOM

## Abstract

Automated assessment and prediction of marital outcome in couples therapy is a challenging task but promises to be a potentially useful tool for clinical psychologists. Computational approaches for inferring therapy outcomes using observable behavioral information obtained from conversations between spouses offer objective means for understanding relationship dynamics. In this work, we explore whether the acoustics of the spoken interactions of clinically distressed spouses provide information towards assessment of therapy outcomes. The therapy outcome prediction task in this work includes detecting whether there was a relationship improvement or not (posed as a binary classification) as well as discerning varying levels of improvement or decline in the relationship status (posed as a multiclass recognition task). We use each interlocutor’s acoustic speech signal characteristics such as vocal intonation and intensity, both independently and in relation to one another, as cues for predicting the therapy outcome. We also compare prediction performance with one obtained via standardized behavioral codes characterizing the relationship dynamics provided by human experts as features for automated classification. Our experiments, using data from a longitudinal clinical study of couples in distressed relations, showed that predictions of relationship outcomes obtained directly from vocal acoustics are comparable or superior to those obtained using human-rated behavioral codes as prediction features. In addition, combining direct signal-derived features with manually coded behavioral features improved the prediction performance in most cases, indicating the complementarity of relevant information captured by humans and machine algorithms. Additionally, considering the vocal properties of the interlocutors in relation to one another, rather than in isolation, showed to be important for improving the automatic prediction. This finding supports the notion that behavioral outcome, like many other behavioral aspects, is closely related to the dynamics and mutual influence of the interlocutors during their interaction and their resulting behavioral patterns.

## Introduction

Behavioral Signal Processing (BSP) [1, 2] refers to computational methods that support measurement, analysis, and modeling of human behavior and interactions. The main goal is to support decision making of domain experts, such as mental health researchers and clinicians. BSP maps real-world signals to behavioral constructs, often abstract and complex, and has been applied in a variety of clinical domains including couples therapy [1, 3, 4], Autism Spectrum Disorder [5], and addiction counseling [6, 7]. Parallel work with focus on social context rather than the health domains can be found in [8, 9]. Notably, couple therapy has been among one of the key application domains of Behavioral Signal Processing. There have been significant efforts in characterizing the behavior of individuals engaged in conversation with their spouses during problem-solving interaction sessions. Researchers have explored information gathered from various modalities such as vocal patterns of speech [3, 4, 10, 11], spoken language use [1, 12] and visual body gestures [13]. These studies are promising towards the creation of automated support systems for psychotherapists in creating objective measures for diagnostics, intervention assessment and planning. This entails not only characterizing and understanding a range of clinically meaningful behavior traits and patterns but, critically, also measure behavior change in response to treatment. A systematic and objective study and monitoring of the outcome relevant to the respective condition can facilitate positive and personalized interventions. In particular, in clinical psychology, predicting (or measuring from couple interactions, without couple, or therapist provided metrics) the outcome of the relationship of a couple undergoing counseling has been a subject of long-standing interest [14–16].

Many previous studies have manually investigated what behavioral traits and patterns of a couple can tell us of their relationship outcome, for example, whether a couple could successfully recover from their marital conflict or not. Often the monitoring of outcomes involves a prolonged period of time post treatment (up to 5 years), and highly subjective self reporting and manual observational coding [17]. Such an approach suffers from the inherent limitations of the qualitative observational assessment, subjective biases of the experts, and great variability in the self-reporting of behavior by the couples. Having a computational framework for outcome prediction can be beneficial towards assessment of the employed therapy strategies and the quality of treatment, and also help provide feedback to the experts.

In this article, we analyze the vocal speech patterns of couples engaged in problem-solving interactions to infer the eventual outcome of their relationship—whether it improves or not–over the course of therapy. The proposed data-driven approach focuses primarily on the acoustics of the interaction; unobtrusively-obtainable, and known to offer rich behavioral information. We adopt well-established speech signal processing techniques, in conjunction with novel data representations inspired by psychological theories to design the computational scheme for the therapy outcome prediction considered. We formulate the outcome prediction as binary (improvement *vs.* no improvement) and multiclass (different levels of improvement) classification problems and use machine learning techniques to automatically discern the underlying patterns of these classes from the speech signal.

We compare the prediction using features directly derived from speech with prediction using clinically relevant behavioral ratings (*e.g.,* relationship satisfaction, blame patterns, negativity) manually coded by experts after observing the interactions. It should be noted that human behavioral codes are based on watching videos of interactions that provide access to additional information beyond vocal patterns (solely relied by the proposed prediction scheme) including language use and visual nonverbal cues.

In addition to evaluating how well directly signal-derived acoustic features compare with manually derived behavioral codes as features for prediction, we also evaluate the prediction of the outcome when both feature streams are used together.

We also investigate the benefit of explicitly accounting for the dynamics and mutual influence of the dyadic behavior during towards the prediction task. The experimental results show that dynamic functionals that measure relative vocal changes within and across interlocutors contribute to improved outcome prediction.

**Fig 1:**
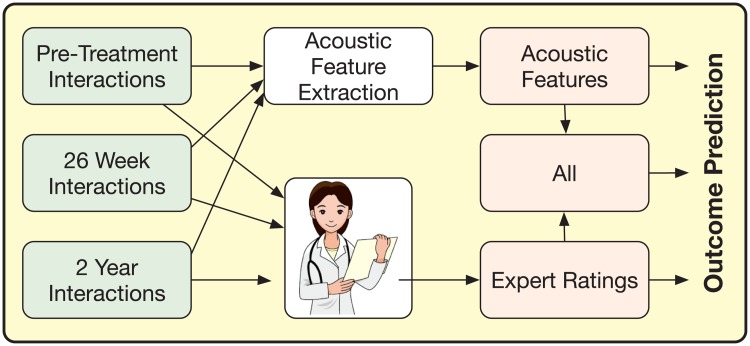
Overview of the work described in this paper. We use 2 out of 3 interactions (shown on left). We employ automated feature extraction from acoustics and/or human behavioral coding (center) and machine learning (right) to derive outcomes.

The outline of the paper is as follows. We discuss relevant literature in Section 1. The Couple Therapy Corpus used in the study is described in Section 1 and illustrated in Fig 1. An overview of the methodologies for speech acoustic feature extraction is given in Section 1 and the use of behavioral codes as features is described in Section 1. We provide an analysis of the proposed acoustic features in Section 1 and the results of the classification experiments in Section 1. Finally, we conclude the paper with a discussion of our findings as well as possible directions for future research in Section 1.

## Related literature

Clinical psychotherapy is an important treatment method for a wide range of psychological problems and disorders including depression, addiction, anxiety, domestic violence and relationship distress. Studies have shown that a typical therapy client is likely to be better off than 75% of the untreated individuals, on average [18].

Over the years, different approaches of psychotherapy have been proposed with methodical differences, but with a shared common goal focused on the personal and social well-being of the individual. In couple therapy, some widely used approaches are Emotionally Focused Couples Therapy (EFCT) [19], Gottman’s Method of Couples Therapy [20], Traditional Behavioral Couples Therapy (TBCT) [21], Cognitive Behavioral Couples Therapy (CBCT) [22, 23], and Integrative Behavioral Couples Therapy (IBCT) [24]. Many studies have compared these different schools of therapy in terms of effectiveness and realizability. Recent works have shown that even though TBCT works well in a short-term basis, IBCT turns out to be the most effective one towards a positive long-term marital outcome [16, 25].

Apart from the inherent nuances of therapy methods, the subjectivity of the therapist and the specific characteristics of the clients can potentially play an important role in therapy. Therefore, it is critical to assess the quality and effectiveness of the therapy process by observing its outcome. Based on this objective, there have been numerous studies on therapy outcomes and comparative analysis of different therapy methods relating to the outcomes. Many of these works focus on the very definition of therapy outcome and the choice of outcome variables by accommodating contextual differences [18, 26–30]. Often, monitoring of outcome over the course of the therapy serves as a good indicator of therapy effectiveness. This has triggered a lot of research on longitudinal outcome studies [31, 32].

Among the different outcome studies, a considerable amount of research has been undertaken in the specific domain of couple therapy, including those that have focused on defining proper metrics for marital therapy outcomes. One of the obvious outcomes, of course, would be the information if the couple stayed in the relationship or not within a certain time after the intervention. However, divorce (or absence of it) does not always reflect the degree of marital satisfaction; whether a couple in a distressed relationship would go through divorce depends on a number of external factors like age, education, culture, religious beliefs and socio-economic status of the spouses [33, 34]. Most of the studies on couple therapy outcomes have focused on outcomes of the couples based on their behavior, either observed from their interactions or through carefully designed questionnaires. One of the first studies of this kind was conducted by Bentler and Newcomb [35], who found a high correlation between certain psychological variables, such as self-perception and other personality traits, reported by the couple through a questionnaire and their marital success. As a general trend of outcome studies in couple therapy, researchers typically have proposed relevant behavioral descriptors of the couple and analyzed how they are related to, and predictive of, marital outcome. Gottman and Krokoff [36] found certain interaction patterns, such as defensiveness and withdrawal, to be detrimental for long-term marital success from the empirical studies they conducted. In [37], the authors have shown codified observed behaviors, such as withdrawal, sadness, and humor, to be indicators of marital success with a cascade representation of possible gradual deterioration with time. Another set of constructed variables, such as disappointment, withdrawal, and fondness, describing the history of oral interviews of the couple were used by Buehlman *et al.* [38]. Another work by Gottman *et al.* [39] received widespread attention for prediction of marital satisfaction and divorce. It also made many recommendations for therapy based on what it deemed beneficial or detrimental for marriage. Other works with similar behavioral coding-based approaches for the prediction of marital success or failure can be found in the literature [40–42]. A comprehensive survey of the marital outcome prediction studies can be found in [43]. Further, two recent books by Gottman [44, 45] have summarized his work on this topic.

In summary, a significant amount research in clinical psychology has sought answer to the question “What leads to a divorce or an unsuccessful marriage?”. Even though these studies have provided important insights into the key factors for marital success, they suffer from certain drawbacks. According to Heyman [46], these shortcomings can range from technical issues like lack of rigorous statistical validation of the hypotheses of the studies to more practical shortcomings such as lack of sufficient reliable data [47]. Another criticism of these studies is that the high prediction accuracy rates reported are often misleading as the experiments were mostly data-fitting analysis instead of prediction with cross-validation and hence subject to overfitting [48]. The limitation of using self-reporting behavioral traits by the couples was highlighted in [49]. Kim *et al.* [50] also argued against the generalizability of these works and highlighted the importance of further research and investigation of behavioral process models of relationship outcomes. Another work [51] also raised concern about some possible methodological flaws in many previous works and in [39] in particular.

A more recent study investigated different factors being responsible for an unsuccessful marriage [52]. It categorized these factors into three categories: demographic (*e.g.,* education), intrapersonal (*e.g.,* depression) and interpersonal (*e.g.,* intimacy, commitment). According to the findings of the hierarchical linear modeling technique used in this work, interpersonal factors have the strongest contribution to the success of a marriage. Moreover, it found that the effect is even stronger during the initial stages of the therapy. In a follow-up of the same study 2 years after the termination of the therapy, communication factors such as encoded arousal (based on pitch), power processes were also included [53]. These communication factors were found to be the strongest predictors of the treatment response after 2 years. Finally, a 5 year follow-up showed that commitment is a key factor behind outcome [54]. This study was based on the Couple therapy corpus [25], which is used in the current work and described in a latter section.

Over the past two decades, psychology and social science have seen a lot of changes in computational aspects coinciding with advances in machine learning, artificial intelligence and more recently fields like *social signal processing* [8, 9, 55, 56] and *behavioral signal processing* [2, 57]. Researchers have shown that *thin slices* [58] or small segments of conversational dynamics can predict interpersonal or behavioral traits or outcomes such as negotiation trends [59], personality [60, 61], depression [62], deception [63, 64], and agreement [65].

In couple therapy, researchers have investigated various signal processing and machine learning based computational methods to study key emotions and behaviors expressed through different modalities of interactions. A majority of these works have used the aforementioned Couple Therapy corpus to validate the signal-driven approaches with real world data. A particularly relevant work on couple therapy is the one that used speech acoustic features to predict different behavioral classes [3, 10], *e.g.,* determining automatically if a person blames his/her spouse during a conversation. Another work [4] analyzed dyadic interaction dynamics, notably the process of entrainment or mutual adaptation of behavior through the course of an interaction and related it to predicting the perceived affectivity. In [1], the authors presented a framework for extracting behavioral information from language use by the couples, while [66] showed the utility of combining speech and language information for behavioral prediction. More recently, dynamic models to characterize the changes in behavior of couples during interactions have been proposed–both in acoustic [67] and lexical modalities [12], and extensions of the lexical work to produce more robust methods have been introduces within a neural-net framework [68]. Finally, some early results from our current work on prediction of marital outcome from acoustic features were presented in [69] with a simpler methodology and basic analyses. In the current work, we developed a improved framework that extracts both short-term and long-term temporal changes in acoustic features.

## Couple therapy corpus and outcomes

The Couple Therapy corpus used in this work is a collection of video recordings of interactions of real couples in distressed relationships. The corpus was collected as a part of a longitudinal study on couple therapy by collaborating researchers from University of California, Los Angeles and University of Washington [25]. The clinical trial that created this corpus primarily focused on analyzing whether Integrative Behavioral Couple Therapy (IBCT) is more efficacious than Traditional Behavioral Couple Therapy (TBCT). To the best of our knowledge, it is also the largest such collection of randomized clinical couple therapy interaction data [25]. All study procedures were approved by the Institutional Review Boards at the University of California, Los Angeles and the University of Washington, written consent was provided by all study participants, and treatment was provided according to the principles of the Declaration of Helsinki.

One hundred and thirty-four chronically distressed couples were recruited to participate in this study. All of them were male-female pairs legally married on average for 10.0 years (*SD* = 7.6). They were also selected after a screening of psycho-pathological conditions that might interfere with the behavioral aspects of interest, such as schizophrenia, bipolar disorder or antisocial personality disorder.

The mean age of the husbands and wives in the study were 43.49 years (*SD* = 8.74) and 41.62 years (*SD* = 8.59), respectively. The majority of the participants identified themselves as Caucasians (husbands: 79.1%, wives: 76.1%); other ethnic groups include African American (husbands: 6.7%, wives: 8.2%), Asian or Pacific Islander (husbands: 6.0%, wives: 4.5%), Latino or Latina (husbands: 5.2%, wives: 5.2%) and Native American/Alaskan (husbands: 0.7%).

The study consisted of three recording sessions collected over a span of 2 years for each couple as illustrated in Fig 1. The first session took place just before the therapy started; the second one was after 26 weeks of therapy and the last session was recorded after two years. However, some of the couples did not follow up and as a consequence, the corresponding post-therapy sessions (26 weeks or 2 years) are missing. Each spouse chose an issue critical to their relationship and discussed it with their partner in each of these problem-solving interactions. The short-term goal of these sessions was the mutual understanding of these conflicting problems and to reach a resolution. Every session again has two parts based on the problem under discussion: whether it was chosen by the husband or the wife. The couples had their interaction in the absence of any therapist or research staff.

***Behavioral Coding: Observational interaction measures by experts***: As a part of the corpus, we also have manually-specified behavioral annotations for each spouse in each session. It was based on observations of the recorded audio-visual interaction of the couple. The behavioral attributes of interest, which we refer to as the *behavioral codes* or simply *codes*, consist of 33 behavioral dimensions combining two established behavioral coding systems: the Couples Interaction Rating System (CIRS, [70]) and the Social Support Interaction Rating System (SSIRS, [71]). These codes are summarized in Table 1. Every session was annotated by multiple (ranging from 2 to 9) human experts and the average of their ratings are used as the reference. For the data we used, the average inter-annotator agreement of these codes in terms of Krippendorff’s *α* [72] measure is 0.7528.

**Table 1:** Behavioral coding systems used in the dataset: SSIRS (Social Support Interaction Rating System) and CIRS (Couple Interaction Rating System).

Coding System	Codes
SSIRS	Global positive affect, global negative affect, use of humor, influence of humor by the other, sadness, anger/frustration, belligerence/domineering, contempt/disgust, tension/anxiety, defensiveness, affection, satisfaction, solicits partner’s suggestions, instrumental support offered, emotional support offered, submissive or dominant, topic being a relationship issue, topic being a personal issue, discussion about husband, discussion about wife
CIRS	Acceptance of the other, blame, responsibility for self, solicits partner’s perspective, states external origins, discussion, clearly defines problem, offers solutions, negotiates, makes agreements, pressures for change, withdraws, avoidance

***Marital Outcome Measures***: The aforementioned couple therapy corpus has been used in a number of research studies on marital outcome in response to different therapies [16, 17, 25]. The two common scales to measure marital satisfaction are the Dyadic Adjustment Scale (DAS, [73]) and the Global Distress Scale(GDS, [74]). Simple comparison of pre-therapy and post-therapy scores using these scales can tell us empirically whether there has been any improvement in the relationship. Couples were categorized into four categories using the formula provided in Jacobson and Truax [75] and a composite relationship satisfaction score based on a combination of the DAS and the GDS. This categorical approach is more interpretable than a continuous score and useful for couples therapy domain since the categories are based on clinically significant change. In psychotherapy, clinical significance of a change is qualitatively defined as the extent to which therapy moves a couple within the control group or functional population. The operational definitions of clinical significance are based on various statistical approaches and are discussed in [75]. The four derived categories are as follows:

- Type 1: **deteriorated** (*i.e.,* they got measurably worse over treatment)
- Type 2: **no change** (*i.e.,* no meaningful improvement)
- Type 3: **improved** (*i.e.,* they got measurably better over treatment, but still clinically insignificant)
- Type 4: **recovered** (*i.e.,* they got measurably better over treatment and their score is above the upper cut-off for clinically significant distress)

These outcome types represented the recovery (or the lack thereof) of the couples at the time of either 26 weeks or 2 years relative to the time they started the therapy. In other words, one such outcome variable is associated with every combination of interaction sessions(*pre*-therapy to *post*-therapy). These outcome ratings will be considered as the reference labels for our automatic classification tasks in this study.

Even though the original corpus had 134 couples, the outcome ratings could not be recorded for some couples due to reasons such as dropout of couples from the study, or lack of sufficient information to rate them. Also the audio quality of some of the recordings was poor. Moreover, some couples had these outcomes labeled only for one of the post-therapy sessions (either after 26 weeks or 2 years). After taking into account all such cases in the dataset, we had 141 instances of outcomes, which included *(i)* outcome after 26 weeks relative to pre-treatment, and *(ii)* outcome after 2 years relative to pre-treatment. Therefore, we have 141 samples in our analyzed dataset, every sample belonging to one of the four outcome classes (with ratings 1 though 4) shown in Table 2. Among these, 53 couples have both outcome variables (26 weeks and 2 years), and 35 couples have only one. There are total 229 recordings with, two 10-minute problem-solving interactions each, resulting in 458 10-minute interactions altogether.

**Table 2:** Number of data samples with different outcome ratings.

Outcome	Decline	No Change	Partial Recovery	Recovery
Rating	1	2	3	4
Count	12	26	34	67

Note that the data comprise of two therapy treatments: Integrative Behavior Couples Therapy (IBCT) and Traditional Behavior Couples Therapy (TBCT). As such, merging them together in a single analysis corpus without exploiting knowledge of the therapy style, is expected to result in a more challenging analysis and more robust models. It introduces no bias, but increases model generalization. We elected to examine only main effects of acoustic parameters for several reasons. First, our interest in this manuscript is on predicting relational outcomes independent of treatment received. Second, and in contrast to earlier work on this corpus, we examined a large number of acoustic parameters. Tests of interactive effects involving more than one acoustic parameter, type of treatment received, and pre-treatment satisfaction are important directions for future research; however, these tests are also meaningfully different research questions than the ones tested in this manuscript. Third, for the purposes of machine learning, robustness can be better achieved through more diverse and larger amount of data.

## Acoustic feature extraction

In this section, we describe the process of acoustic feature extraction from the speech recorded during dyadic conversations. Our aim is to capture relevant cues from the recorded speech acoustic signal relevant to the behavioral outcomes of the speaker in general, and the outcome of the couple therapy in particular. As a starting point, we extracted standard speech features of various kinds including those which are represent both segmental spectral characteristics and prosody. Furthermore, we designed additional meta-features from these standard acoustic features to extract short- and long-term dynamics of the vocal cues of the interlocutors. These meta-features range from turn-level (L1) features within a session to cross-session features (L2). We discuss them in further detail in the following subsections.

### Pre-processing of audio data

In this section, we describe the pre-processing steps employed to prepare the recorded speech data for automated feature extraction and subsequent analysis. We started with all the sessions that we had after the initial screening based on the availability of outcome measures. For every 10 minute session, we had single channel continuous audio recorded from a far-field microphone (16 kHz, 16 bit). Originally the audio was collected with an analog recorder, and digital copies were made prior to processing of the data.

***Voice Activity Detection***: In our study, we focus on acoustic features extracted only for speech regions in the audio recordings of the conversations. For this purpose, we used an automatic Voice Activity Detection (VAD) system as described in [76] to separate the audio stream into speech and non-speech regions. This robust algorithm exploits the spectral characteristics of the audio signal to distinguish speech from background audio. More specifically, it extracts audio features like spectral shape, harmonicity, and long-term spectral variability features with a long duration context window and feeds them to a Multilayer Perceptron classifier. Since we do not have VAD ground truth (manually labeled speech and non-speech regions) for couple therapy dataset, we used the manual transcripts and audio to force-align the text with audio [10] to come up with a proxy for the ground truth. On the evaluation subset of the data, the miss rate of VAD (speech detected as non-speech) was 17.1% and false detection rate (non-speech detected as speech) was 13.6%.

***Speaker Diarization***: Since the speech was recorded continuously with a single channel microphone during a conversation, we need to segment the speech regions belonging to each speaker (the husband’s or the wife’s speech), prior to further speech analyses. To achieve this, we performed speaker diarization in a two-step method: first, the algorithm segments the speech stream based on possible speaker changes using Generalized Likelihood Ratio based criteria in a frame-based analysis, following which speaker-homogeneous segments are clustered using agglomerative clustering [77]. This way we partition the entire interaction session into regions spoken by each of the speakers. We also automatically identified the speakers as husband or wife using their average pitch information [78]. This simplistic approach was adequate since these conversations always involve two people of different genders, and whose pitch patterns tend to be distinct. Based on a performance evaluation similar to VAD, the diarization error rate (DER) was found to be 27.6%. While this error rate for diarization is not satisfactorily low, it might reflect the inaccuracies in the references, which is obtained by automatic speech-to-text alignment. There are also some instances of overlapped speech in the dataset which is not recognized by diarization algorithms.

### Different types of acoustic features

Following the pre-processing steps, we extracted various acoustic features from each of the 458 10-minute sessions, which are already segmented into speaker-specific speech regions and separated from silence regions.

The initial feature extraction is done on a frame-by-frame basis from the audio in every 10 *ms* with a 25 *ms* Hamming window. Pitch, intensity and Harmonics-to-Noise Ratio (HNR) were computed with the *Praat* toolbox [79], while all other features were extracted using *openSMILE* [80]. In total, we used 74 acoustic features in this study, deemed relevant for capturing behavioral information of interest [10], and summarized in Table 3.

**Table 3:** Basic acoustic features used in the study.

Feature Type	Feature Names
Spectral	15 MFCCs and their derivatives, 8 MFBs and their derivatives, 8 LSFs and their derivatives
Prosody	Intensity, Pitch and their derivatives
Voice quality	Jitter, Shimmer, Harmonics-to-Noise Ratio and their derivatives

While a larger number of acoustic features could be derived, given the data sample size we restricted the features to a smaller set that nevertheless captured essential speech properties grouped into three categories: Prosodic features, Spectral features, and Voice Quality features.

***Spectral features***: Even though vocal prosody is more easily interpretable in terms of reflecting emotion and other psychological states of a speaker, speech spectral features are known to encode critical behavioral information [4, 10, 81–84]. In this work, we use 15 Mel-frequency cepstral coefficients (MFCCs), 8 log Mel-frequency band features (MFB) and 8 line spectral frequencies (LSFs). The derivatives of these were also used as features.

***Prosodic features***: Pitch, intensity and their derivatives were the prosodic features used in our study. These features have been of wide interest in psychology research due to the interpretability they afford of the underlying behavioral mechanisms [85–87]. Prior behavioral signal processing research in couples therapy has also validated this through predictive modeling [4, 10, 11, 88]. We used *Praat* [79] to extract pitch (*f*_0_) and intensity, while other prosodic features were extracted using *openSMILE* [80].

***Voice quality features***: Jitter and shimmer are two widely used features for voice quality, and were also considered in this study. Jitter is the short-term cycle-to-cycle variation of pitch, whereas the analogous quantity for amplitude is called shimmer [89]. It has been shown that these capture paralinguistic information and are used emotion recognition [90]. We have also used derivatives of both jitter (also known as jitter-of-jitter) and shimmer. Another voice quality feature that we considered is Harmonics-to-Noise Ratio (HNR) which estimates the noise level in human voice signal.

### Static functionals

Frame-level analysis results in high dimensional data stream both due to the high dimension of features extracted within each frame and the high frame rate. In order to represent the vocal characteristics in a more compact way, often the statistics of the frame-level features such as mean, median and standard deviation are obtained. In this work, we do the same for each of the interlocutors—husband and wife—resulting in two sets of static functionals for every session. As these are computed over one session for every speaker without considering the temporal dynamics or the influence of the other speaker, we call them *static* functionals. This approach is common in most literature looking for session-level attributes from frame-level speech analysis [3, 10, 91, 92].

### Dynamic functionals

Most literature aimed at extracting emotion or other behavioral constructs at a global level from speech relies on using static functionals over the frame-level features or low-level descriptors [10, 82, 91]. This is a reasonable way to reduce the representation overhead of information for high-level inference. Yet, it has been also recognized that due to a high degree of data compression, important temporal information might be lost. This has also motivated some works to employ diverse temporal information of speech features, especially in emotion recognition [93, 94].

Important behavioral patterns are inherently dynamic. For example, dynamic coordination of speech characteristics reflect the psychological states of the interlocutors [95]. In social contexts, they are also reflective of and influential to the nature of social relationships through communicative behavior [96, 97].

This motivates the use of dynamic features that we discuss below. These are designed to be robust and to potentially capture dynamical patterns of speech encoded with behavioral information.

#### Short term dynamic functionals

The acoustic features described in the previous section are based on features of each speaker in isolation, and hence do not fully capture interaction phenomena like dyadic coordination and entrainment. To address this, turn-level analysis is often adopted, for example, in the context of emotion recognition [98, 99]. Lee *et al.* [4] have shown that interlocutors tend to adapt to each other’s behavior during their interaction. This phenomenon, known as behavioral entrainment, is also reflected in speech acoustic patterns and thus motivates the use of features which can capture such coupled changes.

The computation method of short-term dynamic functionals is as follows:

1. The mean of each acoustic feature over each turn of a speaker is computed. This way, every turn taken by the interlocutors is represented by the averaged acoustic features of that turn.
2. Next, we compute the differences (“deltas”) between corresponding features in adjacent turns within and across speakers. So in the dyadic conversation setting of couples, we obtain three types of differences—husband-husband (HH) delta, husband-wife (HW) delta, wife-wife (WW) delta features. One should note that another possible set of functionals, namely, wife-husband (WH) contain the same information, albeit with a reversed sign. Hence they are not considered to avoid unnecessarily increasing the feature dimensionality.
3. Finally, we use the statistical functionals of the turn-level delta features (as listed in Table 4) as short-term dynamic functionals.

**Table 4:** Different features representations used in the study.

Representation	Input	Scope	Definition
Raw features	Audio	25 ms window	as described in Table 3
Static functionals	Raw features	1 session (10 minutes)	Statistics over entire session
Short-term dynamic	Turns	1 session (10 minutes)	Statistics over all turns
Long-term dynamic	Segments	Duration of therapy	Delta between two sessions

**Fig 2:**
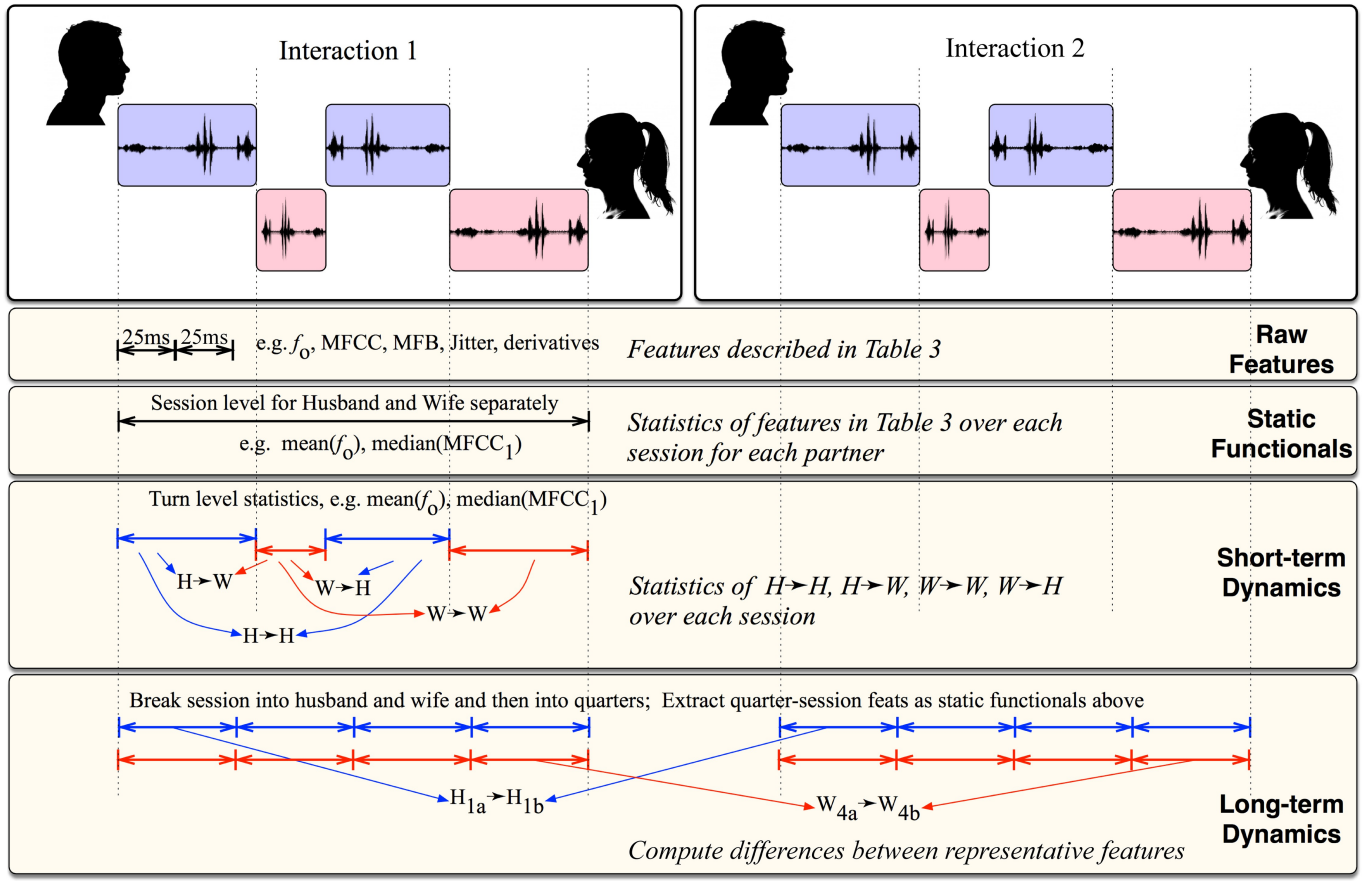
Short-term dynamic functionals capture the statistics of differences between the means of features of adjacent turns in the interaction, both within an interlocutor (*e.g.,* Wife to wife turn changes) but also across interlocutors (*e.g.,* Wife to husband turn changes).

The rationale behind using turn-level measures is that these turn-level differences or *delta* features can capture useful information about the mutual and self-influence of behavioral patterns of the speakers over time within a session. The central idea of turn-level delta features is presented through a schematic in Fig 2.

#### Long-term dynamic functionals

Since we want to extract information about changes in a marital relationship between two different time-points: one before therapy and the other after therapy, we constructed a set of functionals that connects both sessions. They are computed as described below:

1. After removing the silence regions, we split each session into four equal segments.
2. Next, we perform session-level feature normalization by subtracting the mean from each feature and dividing them by the standard deviation, computed over that session. This reduces the effect of any mismatch in the recording conditions between sessions.
3. Then we take the average of every feature over each quarter, separately for the husband and the wife. Each of these average values essentially represents a cumulative sample from the respective quarters.
4. Finally, we compute differences between *representative* features from one quarter in the pre-therapy session and corresponding quarter in the post-therapy session. These represent long-term functionals of the features with respect to pre- and post-therapy sessions.

Conceptually, the design of the long-term dynamic functionals aims to capture two different aspects. Firstly, it captures information from the four quarters of a session thus allowing the features to represent the coarse evolution of dynamics within a session. Second, it captures the direct change in dynamics in sessions before and after therapy.

## Manually-derived behavioral codes as features

In this study, our aim is to investigate whether and how well we can automatically recognize the outcome of marital therapy directly from speech acoustic features of a couple’s interaction. The factors that underlie and influence an outcome such as the relationship status are complex, and multifaceted. It is within this backdrop, we explore what insights automated signal-driven machine-learning approach can offer. We are also interested in investigating how this direct signal-based prediction would compare to a human-driven approach of manually extracting behavioral information and using it for predicting relationship status change post-therapy.

For this purpose, we used the annotations for a set of behavioral codes provided by experts, as described in Section 1. The code set consists of 33 codes in total. All behavioral codes were defined using elaborate guidelines and to be rated on a scale from 1 (“not present”) to 9(“maximally present”). For example, a rating of 8 on the behavioral code for “blame” means the individual was heavily blaming his/her partner during the interaction whereas a rating of 1 means there was no blame at all.

It should be noted that these codes are based on the judgments of the raters using all modalities of interaction present in the video recordings, *i.e.,* speech patterns, facial expression and other gestures, and language information. In other words, these codes are based on both verbal and non-verbal behavior of the couple, made available to the trained annotators.

On the other hand, one limitation of the codes is that since they are each designed for the behaviors of interest for specific research studies, they do not capture the complete behavioral information exhibited by the individuals. Furthermore, they are also affected by subjective bias inherent in human annotations [100].

## Correlation analysis of features with outcomes

After extracting the speech acoustic features and computing functionals of those features, we analyze their relevance to the outcome variable of interest, *i.e.,* the relationship status of the couple. In this section, we present a correlation-based analysis to compare the relevance of different features to the task of inferring the outcome.

We compute Pearson’s correlation coefficient between the outcome and every acoustic feature considered (represented by its static functionals). For this experiment, we have binarized the outcome variable into two classes: recovery (outcome rating 4) *vs.* no recovery (outcome rating 1, 2, and 3 combined). Pearson’s correlation ranges between −1 to +1 and quantifies both the degree and direction of the linear association between the variables. More specifically, a positive value of the coefficient refers to higher levels of one variable being associated to the higher levels of the other, while a negative value represents higher levels of one variable being associated to the negative levels of the other.

In Table 5, we have reported the five most correlated features with the outcome, based on the magnitude of Pearson’s correlation coefficient. In this analysis, for every acoustic feature, we chose the functional with the highest correlation (magnitude); then we compared them for all the features and came up with this list of most relevant features. It should be noted that some of the features are correlated among themselves and thus this list cannot be considered as a sufficient way of identifying the efficacy of the features. However, it provides a straightforward and interpretable way to look into the relevance of the features, to complement the classification experiments that we discuss in following section.

**Table 5:** Pearson’s correlation coefficients of top 5 features and the corresponding functionals (all correlations are statistically significant, i.e., p < 0.05).

Rank	Feature	Category	Functional	Coefficient	p-value
1	MFCC	spectral	mean	−0.2997	0.0003
2	Loudness	prosodic	std. dev.	0.2983	0.0003
3	MFB	spectral	median	0.2859	0.0005
4	Jitter	voice-quality	mean	−0.2791	0.0006
5	Pitch delta	prosodic	mean	0.2772	0.0008

Moreover, we perform a two-tailed significance test of correlation to determine if the these correlations are statistically significant. More specifically, we tested against the null hypothesis that the corresponding feature is not correlated with the binary outcome variable. For all the features mentioned in Table 5, *p* < 0.001 is obtained, which indicates significant correlation.

**Fig 3:**
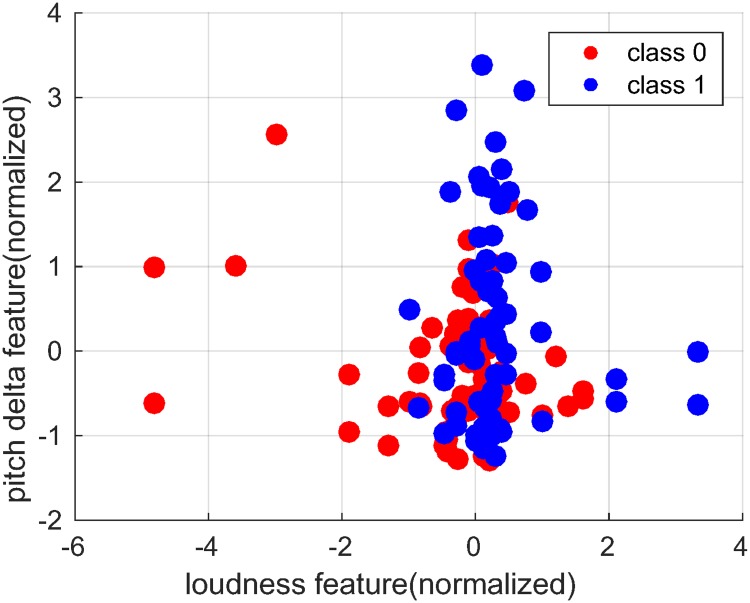
Scatter plot of two prosodic features(normalized) with highest correlation: Loudness (r = 0.2983) and pitch delta (r = 0.2772). The corresponding static functionals are standard deviation and mean, in respective order. Class 0 and class 1 represent respectively no recovery and recovery cases.

In Fig 3, we show the scatterplot of two prosodic features (normalized) with highest correlation coefficient values: standard deviation of loudness (*r* = 0.2983) and mean pitch delta (*r* = 0.2772). From the plot (as well as the positive sign of correlation coefficients), one can infer that high changes in pitch (*i.e.,* high values of mean pitch delta) and a high variation in loudness (*i.e.,* high values of its standard deviation) are associated with a positive outcome.

## Classification experiments

The goal of our classification experiments is to investigate the possibility of inferring distressed couple’s marital outcome using speech patterns of their interaction. As mentioned in Section 1 and shown in Table 2, the outcome can be of 4 defined ratings [1–4]. It should be noted from Table 2 that different number of couples belonging to different outcome classes create a large imbalance, which affects the performance of most classification algorithms [101]. So, we decided to conduct multiple classification experiments, which are listed below:

**Experiment 1**: Classification of all data samples into 2 classes, *i.e.,* complete recovery (rating 4) *vs.* incomplete or no recovery (ratings of type 1, 2, 3 combined)
**Experiment 2**: Classification of instances of no (or incomplete) recovery into finer levels, *i.e.,* rating 1 *vs.* rating 2 *vs.* rating 3
**Experiment 3**: Classification of each possible outcomes *i.e.,* ratings 1 through 4.

As the number of classes increases from Experiment 1 to Experiment 3, the difficulty of the classification also increases—Expt. 3 > Expt. 2 > Expt. 1.

### Experiments with different feature sets

For each of these aforementioned experiments, we investigate the performance of various feature sets extracted from pre- and post-therapy sessions:

1. acoustic features with static functionals,
2. acoustic features with dynamic functionals (both short-term and long-term),
3. acoustic features (with all functionals),
4. manually(human)-derived behavioral codes as features,
5. all features (acoustic features with all functionals and behavioral codes combined)

For each of the classification tasks, we perform z-score normalization on every feature and use a feature selection method to select an optimal subset of features. Also, to account for variability in the dataset, a 10-fold cross-validation is performed. While generating the cross-validation subsets, two post-therapy sessions from the same couple (after 26 weeks and 2 years) are always put together in a single subset (either training or test). In this way, we ensured that there was no data contamination between the training and test datasets.

### Classifier

We set up the prediction problem as three different classification problems and use the well-known *Support Vector Machine* (SVM) algorithm for all three. SVM is a binary classifier by origin, yet it has been later extended to solve multi-class problems and been shown to perform well [102]. In these multi-class problems, we used the one-against-all method, which, as the name suggests, decomposes the multiclass problem into a number of binary classification problems. Throughout all experiments we used the radial basis function (RBF) kernel. Standard parameters of RBF kernel SVM, namely *C* and *γ* were optimized by a simple grid search, separately for each feature set and each experiment. As an example, C = 1000 and gamma = 0.001 were optimally chosen for Expt. 1 with all features.

### Feature selection

The feature extraction (Section 1) leads to a high dimensional feature set, particularly compared to the sample size of training data available. We perform feature selection to choose a subset of the original features that provides the maximum information in the context of a particular classification problem. We consider two feature selection approaches in this work. First, we use a simple correlation-based feature selection method, where we ranked all features using Pearson’s correlation coefficient (discussed earlier in Section 1) as the selection criteria. Next, we also use the Mutual Information Maximisation (MIM) [103] feature selection method available as a part of the *FEAST* toolbox [104]. In this method, every feature *X*_*k*_ is given a *mutual information score* with respect to the class label *Y* as follows:

$$\begin{array}{c} J_{\text{MIM}}=I\left(X_{k};Y\right) \end{array}$$ (1)

Features with the highest *mutual information scores* are selected and the optimal number of features is also determined using cross-validation. We obtained better prediction results using MIM method and decided to utilize it for all the subsequent experiments.

### Results

Table 6 shows the classification accuracy of different feature sets using SVM as the classifier. In the table, mean accuracy and standard deviation over all cross-validation folds are reported for each setup. In addition, the original dimensionality of each feature set is also reported. Every feature set was reduced by using feature selection prior to actual classification. For different experiments, around 10% to 20% of the original features were selected by feature selection. The first row contains the accuracy by chance, computed as the percentage of samples belonging to the largest class.

**Table 6:** Classification accuracy (in terms of their mean and standard deviation over all folds of cross-validation) of different experiments (across the columns) with different feature sets (across the rows).

Featureset	Dim.	Expt. 1		Expt. 2		Expt. 3	
		*mean*	*SD*	*mean*	*SD*	*mean*	*SD*
Chance	-	51.8	-	47.2	-	48.2	-
Behavioral codes	264	75.6	13.5	65.4	14.7	61.8	11.2
Static functionals	3552	76.4	10.0	70.9	13.8	63.2	11.4
Dynamic functionals	6696	78.9	7.6	71.1	12.8	61.5	12.3
Acoustic (all functionals)	10248	79.3	10.2	72.6	13.0	**64.1**	12.8
All features	9144	**79.6**	7.4	**74.6**	12.6	64.1	13.2

As our dataset is highly imbalanced (especially for the multiclass classification), we also computed F-measures [105] of the predicted labels for each setup. The mean and standard deviation of F-scores over all cross-validation folds are shown in Table 7. By definition, the F-measure values lie in the interval (0, 1). A higher value of F-measure signifies better quality in classification.

**Table 7:** F-scores(in terms of their mean and standard deviation over all folds of cross-validation) of different experiments (across the columns) with different feature sets (across the rows).

Featureset	Expt. 1		Expt. 2		Expt. 3	
	*mean*	*SD*	*mean*	*SD*	*mean*	*SD*
Behavioral Codes	0.68	0.12	0.49	0.11	0.48	0.11
Static functionals	0.56	0.10	0.60	0.07	0.52	0.09
Dynamic functionals	0.63	0.05	0.59	0.07	0.50	0.09
Acoustic (all functionals)	0.70	0.09	0.64	0.08	**0.57**	0.11
All features	**0.78**	0.07	**0.64**	0.09	0.56	0.10

There are several observations to make from the obtained classification accuracy and F-measures. First, in general classification based on speech acoustic features tends to outperform the one with behavioral codes extracted by human experts. Specifically, acoustic features (with all functionals) outperformed behavioral codes in terms of accuracy by 2.1% in Expt. 1, 6.9% in Expt. 2, and 1.6% in Expt. 3 (absolute). It is encouraging to see that using acoustic features directly derived from the signal can capture useful information relevant to predicting couples’ relationship status, better than even domain experts can via the manually coded behaviors.

Comparing the different acoustic features, we observe that dynamic functionals perform better than static ones in Expt. 1 and 2. In Expt. 3, however, static functionals achieved better accuracy. The significance and complementarity of both can be seen through the use of all the features.

The results of fusing manual rating based features and acoustic features are mixed. While fusion appears to help in classification in Experiments 1 and 2, we obtain lower accuracy in Experiment 3. We believe the reason for this might be due to overfitting of some behavioral features. For this experiment, the training accuracy (averaged over cross-validation folds) using all features is 73.4%, about 9% higher than the accuracy on the test subsets. This indicates that it is possible that some behavioral codes were selected by the feature selection algorithm from the combined feature set as it helped to achieve low accuracy in training subsets of cross-validation, but it failed to do so in the test subsets. Moreover, issues like the data imbalance and data sparsity become more prominent in Experiment 3 due to the higher number of classes. Another possible explanation for this pattern of findings is that Experiment 3 involves prediction of both changes in and levels of relationship satisfaction while Experiments 1 and 2 involve prediction of only changes in relationship satisfaction. Previously published work on this corpus [53] has found that associations between acoustic features and levels of relationship satisfaction depend on wives’ pre-treatment relationship satisfaction and on the type of couple therapy a couple received. The type of couple therapy and wife pre-treatment relationship satisfaction, although known, were not considered in the analyses in the current paper. It is possible that introducing this additional prior knowledge could help further.

We also perform a two-tailed exact binomial test [106] to verify whether the difference in classification results of different feature sets (reflected in accuracy and F-score measures) is statistically significant. In particular, our null hypothesis is that the results of two feature sets in each test are not significantly different from each other. The *p*-values are reported in Table 8. We observe that using acoustic features produce significantly different results in comparison to using behavioral codes. The differences in performance of all acoustic features (including dynamic functionals) *vs.* static functionals only are significant as well. Finally, in most cases, combining acoustic features and behavioral codes make significant difference in performance, which indicate presence of complementary information in behavioral codes and acoustic features. The only exception is all features combined *vs.* acoustic feature set with all functionals for Experiment 3. In addition, we report the 95% confidence intervals of the statistic computed in each hypothesis test using Clopper-Pearson’s method [107] in Table 9. As we can observe, the confidence intervals are narrow in most cases.

**Table 8:** p-values of statistical significance test against the null hypotheses that the there is no significant difference in performance of the two feature sets compared. The entries in **bold** indicate statistically signifcant difference (p < 0.05).

Comparison	Expt. 1	Expt. 2	Expt. 3
Acoustic (all) *vs.* Behavioral Codes	**0.016**	**0.028**	**0.027**
Acoustic (all) *vs.* Static	**0.034**	**0.042**	**0.039**
All features *vs.* Behavioral Codes	**0.013**	**0.008**	**0.025**
All features *vs.* Acoustic (all)	**0.025**	**0.045**	0.079

**Table 9:** 95% confidence intervals of the statistic for significance test for comparing different feature sets.

Comparison	Expt. 1	Expt. 2	Expt. 3
Acoustic (all) *vs.* Behavioral Codes	(0.019 0.243)	(0.284 0.395)	(0.159 0.271)
Acoustic (all) *vs.* Static	(0.276, 0.294)	(0.221, 0.258)	(0.376, 0.457)
All features *vs.* Behavioral Codes	(0.009 0.133)	(0.156 0.237)	(0.184 0.208)
All features *vs.* Acoustic (all)	(0.240, 0.303)	(0.298, 0.334)	(−0.029, 0.311)

The software employed in this work can be found at http://scuba.usc.edu/software.

## Conclusion

In this article, we presented a study on automatically predicting the marital relationship status of distressed couples in therapy using acoustic information from their speech. We presented a framework for capturing behaviorally significant acoustic features from the spoken interactions of couples engaged in problem solving discussions. We also introduced knowledge-driven features of capturing short-term and long-term acoustic descriptors inspired by previous studies on human interactions. We compared this automatic approach of capturing important behavioral information directly from speech signal to the traditional approach taken by psychologists, *i.e.,* manual coding of behavior from therapy sessions.

In the multiple classification experiments, we observed that the acoustic features from speech capture more relevant information than the manually constructed behavioral dimensions for predicting the marital outcomes from human experts. This is a promising finding considering the fact that human coders had utilized multiple modalities (speech, visual and lexical information) in their coding process. Even though behavioral codes are not designed to predict outcomes itself, they function as behavioral descriptors of the couple and one can expect them to be informative towards the outcome based on the observational methods of psychology.

We also found that dynamic functionals are better than traditional static functionals of acoustic features for outcome prediction. This work opens up avenues for many other research applications and similar frameworks for various behavioral outcome prediction tasks such as assessing results of treatment for various disorders and conditions.

In the future, we can also analyze the importance of other communication modalities including language use (*i.e.,* what is being spoken), and visual (*e.g.,* head-movement and other face and body expressions). One can also investigate more complex temporal modeling (*e.g.,* hidden Markov models, dynamical systems modeling) of the behaviors captured through the acoustic features extracted from the speech signal. Also, automatic recognition of the mental states (such as emotional arousal) of the speakers and investigation of the dynamics of local behavioral cues might be useful.
